# Surgical Ventricular Restoration: An Operation to Reverse Remodeling - Clinical Application (Part II)

**DOI:** 10.2174/157340309789317913

**Published:** 2009-11

**Authors:** Ganesh Shanmugam, Imtiaz S. Ali

**Affiliations:** Department of Surgery, Division of Cardiac Surgery, Dalhousie University, QEII Health Sciences Centre, Halifax Infirmary, 1796 Summer Street, Halifax, Nova Scotia B3H 3A7, Canada

**Keywords:** Surgical ventricular restoration, remodeling, heart failure.

## Abstract

The first part of the article dealt with the basic science behind the evolution of ventricular restoration procedures and the rationale for the use of novel surgical techniques. The second part describes the preoperative workup of patients in advanced heart failure, the core information required to determine the surgical approach and the essential principles and techniques of ventricular restoration. It then examines the effects of ventricular restorative procedures on pump function and clinical outcomes, the results of the worldwide experience with ventricular restoration and concludes with more recent advances in this field.

## 1. PREOPERATIVE WORKUP

A comprehensive preoperative evaluation for surgical planning includes: (a) measurement of the asynergic (noncontracting) area of the scar, which is the nidus for remote dilation, (b) determination of the adequacy of remote muscle to support the circulation after scar exclusion, (c) ventricular volumes and indices, (d) viability of the surgical target and the remote muscle, (e) regional and global function, (e) mitral annular dilation, (f) interpapillary muscle width and (g) quantitative assessment of wall motion by tagging. Cardiac magnetic resonance imaging (MRI) allows a complete evaluation of these quantities [1].

1. The scar is the nidus for subsequent dilation of remote muscle and asynergy >35% of the ventricular perimeter by ventriculography generally causes LV dilatation. Contiguous asynergy > 25% is amenable to SVR. The region to be surgically excluded is evaluated for wall motion and thickening. Late contrast enhancement helps characterize both infarcted and remote muscle. The region of asynergy whether dyskinetic or akinetic lacks contractility. Viability of the asynergic region (<50% transmural late enhancement) predicts functional recovery with revascularization alone, while predominant non-viability (>50% enhancement) may preclude contractile recovery following revascularization alone. SVR excludes this nidus for dilation and reduces LV volume in such cases.
2. Tagging labels a specific volume of tissue whose motion is tracked through the cardiac cycle. Local myocardial contractility can be determined by the distortion of the tagged element boundaries. This is useful when a region of viable, contractile myocardium is adjacent to and tethers a region of nonviable, noncontracting muscle, thereby making the nonviable region appear contractile. Tagging clarifies this issue, since a viable, contracting segment exhibits ‘squeezing’ deformation of tagged tissue, while a nonviable segment that is tethered shows a ‘stretching’ deformation.
3. Remote muscle function provides the compensatory element for survival. Documentation of remote muscle contractility, or its viability if hypokinetic predicts functional recovery after SVR. The remote myocardium could be normal, infarcted, stunned or hibernating. Hibernating muscle (due to right or circumflex coronary stenoses), if inadequately revacularized, may lead to pump failure after SVR, if contractility is inadequate to support circulatory needs.Scarring of remote myocardium determines prognosis and is critical in patient selection. Remote muscle viability helps decide if revascularization of the circumflex or right coronaries is likely to be beneficial, as extensive scarring may preclude contractile recovery.
4. Viability analysis by MRI includes assessment of regional function and morphology. Myocardium with diastolic wall thickness < 5.5 mm may be nonviable.

## 2. SVR PRINCIPLES

The fundamental principle of SVR is to exclude dead muscle and revascularize damaged remote muscle. This restores contractile capacity of the dilated hibernated remote muscle, which compresses the intraventricular patch, thereby improving mechanical performance. Evidence of patch movement has been observed by echocardiography and colour kinetics. The patch acts as an active endocardium and no significant dysfunctional zone is evident. LAD grafting is important to revascularize the upper septum. RCA/LCX grafting is crucial to revascularize the remote muscle which provides postoperative pump function [2].

## 3. SURGICAL TECHNIQUE

The concept of SVR consists of complete revascularization to relieve ischemia, ventricular reconstruction to restore elliptical shape, reduction of volume and wall stress to improve hemodynamics and when necessary, endocardectomy and cryoablation for VT [3-5]. Mitral valve repair is performed as needed.

### Operative Procedure

Following initiation of cardiopulmonary bypass and cardioplegic arrest, coronary revascularization and mitral repair are performed as necessary. With fibrotic, dyskinetic LVA, there is a clear transitional zone between dysfunctional tissue and viable contracting myocardium. Traditionally a linear repair technique has been used on thin-walled left ventricular aneurysms but this does not allow for septal exclusion. Lundblad [6] demonstrated that surgical risk is lower and long  term survival is higher after endoventricular circular patch plasty [EVCPP] than simple linear repair.

The LV is opened through the infarcted, scarred area, which often collapses on cardiopulmonary bypass and venting. The ventriculotomy is extended parallel to the LAD. Clots are removed. A subtotal nonguided endocardectomy is performed on the septum and anterior wall if spontaneous or inducible VT exists. Linear cryo lesions are applied at the edge of the endocardial resection.

Akinetic areas must be recognized. With post-MI revascularization, the classic myocardial scar that collapses on venting may not be evident. It is important to exclude the entire segment to delete the midmyocardial and endocardial scar.

SVR can be performed on an arrested or beating heart. SVR on a beating heart has distinct advantages in thick walled akinetic ventricles where visual demarcation between scar and healthy muscle is not obvious.

Menicanti [7] introduced the use of a mannequin filled at 50 to 60 ml/m^2^ to optimize size and shape of the new ventricle. The mannequin is useful when the ventricle is not much dilated (to reduce the risk of a small residual cavity) or when the infarct is not clearly demarcated. Mannequins therefore prevent excessive reduction of ventricular volume, help position the new apex, create an elliptical shape and provide adequate volume reduction in huge ventricles where the scar is not readily apparent. Creation of a small LV results in a low cardiac output state, while a residual box like LV is characterized by diastolic dysfunction. Residual sphericity causes papillary muscle malalignment and MR.

An endoventricular circular suture (Fontan stitch) is placed at the transitional zone starting from the new apex, going medially toward the septum, superiorly toward the aortic valve, laterally toward the base of the papillary muscles and ending at the  level of the apical reference stitch. The Fontan stitch is tightened to approximate the ventricular wall to the balloon.

A Dacron or pericardial patch is sewn to the ridge created by the purse-string suture. The ventriculotomy is then folded over the patch and closed. Patients are weaned from cardiopulmonary bypass with inotropes and/or an intraaortic balloon pump (IABP) as necessary.

Retention of spherical shape may persist when patch placement is limited to the scar, since extensively stretched remote muscle stretch is retained. This results in a spherical LV, with a box like apex on post-operative angiograms. This is the pitfall of using the scar as the guide in grossly dilated ventricles.

The SAVE or Pacopexy procedure [8] was initially developed for non-ischemic cardiomyopathy and is based on selecting the site for muscle resection depending on intraoperative echo findings, since muscle involvement is heterogenous. It was developed to reconstruct elliptical form, with patch placement in an oblique direction between the apex and the septum just below the aortic valve [9]. Patch placement differs from the Dor procedure where the scar defines patch placement.

#### 3A. Akinesia *vs*. Dyskinesia

Di Donato *et al. *demonstrated that outcome is more strongly linked to the extent of asynergy than to the type of asynergy (akinetic vs dyskinetic) [10]. Once the infarcted scar exceeds 20% of the LV surface area, cardiac function declines. Both dyskinetic and akinetic scars share similar mechanical defects.

#### 3B. Site Selection

In advanced idiopathic dilated cardiomyopathy, the extent of interstitial fibrosis is heterogenous. The choice of procedure is assessed by preoperative echocardiography, cardiac MRI and an intraoperative volume reduction test to find the weakest area.

The intraoperative echocardiography-guided volume reduction test helps detect changes in wall motion and thickness, when the dilated LV is decompressed on bypass, with a consequent reduction in wall tension [11]. The SAVE procedure [Pacopexy] is performed to exclude a weak septum. PLV, as described by Batista is used with predominant lateral wall involvement

Consequently, the intraventricular guideposts for reconstruction that usually involves patch placement may alter from current attention that is directed only at the visible scar to the creation of a more natural form that may place sutures beyond the obvious disease.

## 4. MITRAL INVOLVEMENT

Functional MR results from LV dysfunction and remodelling, LV sphericity, incomplete leaflet closure, papillary muscle displacement and systolic valvular tenting. MR portends a worse prognosis and overestimates remote muscle function. The dysfunctional LV is intolerant of any degree of MR greater than moderate.

Local deformation of the inferior wall with loss of systolic inward bending is associated with functional MR. End-systolic inferior wall curvature is a key determinant of mitral valve closure. If the inferior wall has a normal negative curvature (i.e., convex toward LV cavity), the mitral leaflets coapt normally. If the curvature is not negative (i.e., flat or concave), mitral tenting ensues. The inferolateral wall is the continuity between the basal loop and the descending segment of the ascending loop and explains the functional impairment of a region remote from the ischemic damage. Asynergy and systolic deformation of the inferobasal region and high capillary wedge pressure are prognostic signs of MR development late after endoventricular circular patch plasty repair [12].

Mitral annuloplasty with an undersized ring was proposed by Bolling [13]. Undersizing mitral annuloplasty has shown favorable results because of its low operative risk with symptomatic relief but its effect on longevity is controversial [13]. Patients with dilated ventricles show poor outcomes after mitral reconstruction alone, demonstrating the need for ventricular reduction.

The amelioration of mild mitral insufficiency after the Dor procedure relates to the restoration of LV ventricular geometry which results in a narrower configuration of papillary muscles. Improvement in functional MR is observed in 64% of patients despite no mitral intervention.

The presence of moderate-to-severe MR and its surgical repair carries a higher operative risk. MR becomes a true predictor of hospital mortality only when the LV end-diastolic pressure, and therefore the left atrial pressure are severely increased, leading to CHF and severe functional impairment (NYHA class III/IV).

When the degree of MR is moderate to severe or the  annulus is dilated and ventricular function is  severely depressed, the mitral valve should be preferably repaired during the SVR procedure.  Di Donato and colleagues [10] demonstrated that late MR after the Dor procedure is associated with poor prognosis and emphasized early (intraoperative) correction.

Paradoxically, in a substantial number of patients without MR preoperatively, MR develops 1 year later. To prevent the risk of late MR, a more than 30ml/ m^2^ reduction of enddiastolic volume index should be avoided. Patients with late MR generally have greater preoperative volumes than did patients without late MR.

## 5. SVR AND VENTRICULAR TACHYCARDIA

Sustained VT is associated with increased mortality in ischemic cardiomyopathy. Monomorphic premature ventricular contractions originating from the scar border zone can be triggers for ventricular fibrillation. Coronary artery bypass graft surgery alone does not eliminate the risk of ventricular arrhythmias [14]. In 1994, Dor and associates presented an addition to the original endoventricular circular patch plasty, for patients with VT and a success rate exceeding 90% in curing VT was achieved [4]. The Dor procedure eliminates the need for an implantable defibrillator in most patients. However in cases with postoperative VT, an ICD is recommended.

Mapping studies by Mickleborough [15] in patients with recurrent VT showed that ease of arrhythmia induction was related to mechanical loading conditions. Myocardial stretch is arrhythmogenic. Late recurrences of ventricular arrhythmias after LV reconstruction might be due to progressive remodeling and LV enlargement.

## 6. THE EFFECTS OF SVR 

SVR alters ventricular shape and volume, improves survival, functional class, and ejection fraction [3, 10, 16] and can achieve a high degree of freedom from re-admission for heart failure. It creates a mechanical intraventricular resynchronization in patients without conduction delay, which improves LV performance and may reduce ventricular arrhythmias in the dilated heart [17]. Recent reports [18] indicate a higher mortality when preoperative LVESVI > 120 ml/m^2^ as a larger chamber is left if exclusion is limited to the edge of the scar.

Tulner and colleagues [19] used conductance catheters to show that SVR normalized left ventricular volumes, reduced left ventricular wall stress and oxygen consumption and improved wall compliance and mechanical efficiency.

### 6A. Effect on Systolic Function

The relatively small changes in systolic function in patients who had isolated restrictive mitral annuloplasty or CABG indicate that the systolic improvements following SVR are mainly related to LV restoration. The increase in EF is attributed to a reduction in end-diastolic volume, as LV stroke volume is unchanged. However, EF is an inaccurate index of systolic performance after SVR due to altered post-operative loading conditions. The slope of the end-systolic pressure-volume relation, end-systolic elastance E_ES_, is a load-independent index of systolic function and improves significantly after SVR. Schreuder [20] used cardiac catheter data to document an improvement in E_ES_ after the DOR procedure. This improvement is induced by exclusion of a large compliant scar, and recruitment of remote myocardium.

### 6B. Effect on Diastolic Function

Resection of a portion of the LV wall can detrimentally affect diastolic chamber properties and (2) the effect of such a procedure on overall pump function reflects the balance of its effects on systolic and diastolic properties [21]. The effects on diastolic function are more controversial and complex to interpret. The relaxation time constant τ is significantly reduced, indicating faster relaxation. This time-constant quantifies the speed of LV pressure decay during isovolumic relaxation [19]. This change may result from coronary revascularization—which may enhance the oxygen-dependent reuptake process of calcium by the sarcoplasmic reticulum or from an afterload reduction as active relaxation is afterload dependent.

The LV relaxation indices −dP/dt_max_ and τ are generally decreased and prolonged respectively, in congestive heart failure and in LV aneurysm, which indicates LV mechanical dyssynchrony in early diastole [22]. After patch aneurysmectomy −dP/dt_max_ was lower and τ was markedly reduced, indicating an improvement in LV relaxation during early diastole. Overall LV diastolic function however appeared to be compromised. The diastolic pressure-volume relation was significantly shifted toward smaller volumes and tended to be steeper, as evidenced by an increased diastolic stiffness constant.

### 6C. Effect on Work 

SVR induces a leftward shift of both the end-systolic and the end-diastolic pressure-volume relations. Stroke work and cardiac output are maintained but at the expense of a higher end-diastolic pressure after SVR, indicating reduced reserves to further augment stoke work by the Frank-Starling mechanism [19]. Recurrent LV remodelling can occur especially in patients with a larger preoperative LVESVI.

### 6D. Effects on BNP

Brian natriuretic peptide [BNP] is a hormone released from the ventricles in response to wall stress and ischemia. SVR induces a persistent reduction of NT-pro-BNP and BNP 6 months postoperatively and at late follow-up respectively. Increasing ejection fraction and decreasing LVESV correlated significantly with lower BNP and NT-pro-BNP levels 6 months after surgery [23]. No significant linear association between peptide levels and ejection fraction or LV volumes was found at baseline.

## 7. CAUSES FOR FAILURE

The return of heart failure after the surgery is attributed to (1) recurrent MR, (2) continued LV remodelling; (3) too small a LV caused by excessive excision, (4) removal of the kinetic area while retaining poorly contractile and more fibrotic regions, (5) evolution of coronary artery disease or repeated ischemic episodes and (6) improper identification of the transition between scar and normal muscle in akinetic regions, leading to possible salvage of an excess of scarred ventricle.

## 8. SVR DATA

Maxey [24] and Ribeiro [25] showed that outcomes following CABG + SVR were superior to CABG alone in patients with ischemic cardiomyopathy. Most reported studies are not randomized and the question as to whether adding SVR to coronary artery bypass grafting (CABG) will improve survival and clinical status will be answered by the ongoing Surgical Treatment of Ischemic Heart failure Trial [STICH trial] [26].

## 9. STICH TRIAL 

The STICH trial will address the management of patients with heart failure who have CAD amenable to revascularization. This is a prospective randomized study with 2800 patients from 100 centres. Patients with left ventricular dysfunction and coronary artery disease amenable to CABG will be randomized to combinations of three different treatment strategies: CABG, SVR, and intensive medical therapy (MED).

- The revascularization hypothesis states that coronary artery bypass grafting (CABG) with intensive medical therapy (MED) improves longterm survival compared to MED alone.
- The reconstruction hypothesis states that in patients with anterior LV dysfunction, surgical ventricular restoration (SVR) to a more normal LV size improves survival free of subsequent hospitalization for cardiac cause in comparison to CABG alone.

When compared with MED alone, CABG is expected to confer a 20% reduction in mortality. These data are expected to solidify the role of bypass surgery in the treatment of ICMPY. Ventricular restoration is also expected to improve hospitalization-free survival by 20% when compared with CABG alone. This will also serve to dramatically improve outcomes in a patient population that has a 50% 3-year survival rate without SVR.

The STICH protocol uses LVESVI measurement to make decisions about placing patients into the 3 study groups. If LVESVI is less than 60 ml/m^2^, the intervention decision relates to comparing the effects of either using medical treatment or adding CABG. When LVESVI > 60 ml/m^2^, the decision question relates to how SVR fits into the treatment scheme in 1200 patients.

## 10. RESULTS 

Contemporary results following SVR and the risk factors for mortality are included in Table **1**. An analysis of the use and outcomes of SVR in a national sample in a real world application reveals that perioperative events are somewhat higher than prior selected series.

The performance of SVR in patients after a recent MI or in an emergent setting is contrary to what was done in RESTORE group, where the surgery was performed an average of 4.4 years after an anterior infarct. However, Di Donato *et al.* [27] have demonstrated an operative mortality of 5.4% in 74 patients with an SVR performed within 30 days from an anterior infarct [16]. The patients in the Di Donato et al study did not wait the typical 3 months for SVR that is standard at their center because of angina, NYHA class IV symptoms, heart failure, arrhythmias and cardiogenic shock. Performing SVR after a recent MI, makes it difficult to appreciate the limits of remodelling, but provides the benefit of early revascularization and the early exclusion of akinetic myocardium thereby avoiding subsequent remodelling. Conte *et al.* showed excellent functional outcomes in such patients, with nearly ¾ of the patients improving from NYHA class III/IV to NYHA class I or II following surgery.

## 11. RECENT ADVANCES and CONCLUSION

The effects of shape restoration may be combined with cell implantation into remote muscle [28] to enhance recovery of retained diseased muscle. Heart failure secondary to post-infarction left ventricular remodeling can be reversed by SVR. Surgical ventricular restoration by the Dor procedure can achieve good long-term survival and a high degree of freedom from readmission for heart failure in patients with advanced ischemic heart disease.

## Figures and Tables

**Table 1:** Results of Surgical Ventricular Restoration

No.	Author	Ref..	Pt no	Comments	EF Pre/Post op %	LVESVI and LVEDVI Pre/Post op ml/m2	OM % Early/late	OM % Elective/emergency	Freedom from ccf, card death Rehospitalisation%	Survival % 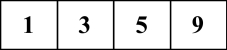	Conclusions	Risk Factors
1	Suma	[29]	95	NYHA ¾ patients 36% pre op inotropes Selected ventriculoplasty + mitral annuloplasty	22 to 27 CI Pre/Post op l/m2/min 2.3 to 2.8		11.6% 27 late	6.6 *vs*. 31.6	22 late card deaths IABP -24 pts LVAD – 2 pts	Overall 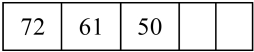 inotropes 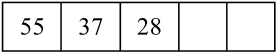 No inotropes 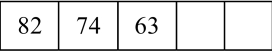	Better results with mitral annuloplasty than replacement Surgery preferably before inotropic dependency NYHA dec from 3.5 to 1.7	Preoperative inotropes NYHA 4 High BNP MVR *vs.* annuloplasty Inc PAP
2	Sartipy	[23]		Measured changes in BNP after SVR	24 to 37 CI Pre/Post op l/m2/min 2 to 2.5	75 to 52 110 to 90						
3	Sartipy	[30]	136				7.4		78 & 58 @ 1 & 5 YRS	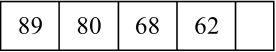		Previous cardiac surgery – early OM Age, IDDM, MR ¾ -late mortality Age, severe MR-rehospitalization
4	Dor	[31]	1150				7.5			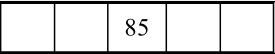		
5	Menicanti	[5]	985				7.2					
6	RESTORE	[3]	1198	Reconstructive Endoventricular Surgery returning Torsion Original Radius Elliptical shape to the left ventricle	29 to 39	80 to 56	5.3		78% @ 5 yrs	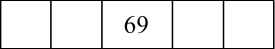 3 YR survival 80 *vs.*65 in NYHA 3 *vs.* 4. 8.7% *vs.* 4 % repair *vs.* no repair NYHA 67% in ¾ 85% in 1/2 Iabp -8.2%		Mitral valve repair EF < 0.3 ESVI > 80 ml/m2 Advanced NYHA class Age > 75 years
7	Mickleborough	[32]	285				2.8			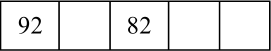		EF < 0.2 CHF Preoperative VT Hypertension
8	Di Donato	[18]	245	61% had CCF 54% were NYHA 3/4			8.1			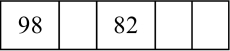		NYHA class EF ESVI Remote asynergy
9	Sartipy	[30]	136				7.4		58% @ 5 yrs	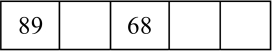		Redo cardiac surgery Age Diabetes MR grade III–IV
10	Isomura	[33]		SAVE * vs.* DOR	23 to 31	135 to 96		4 *vs.* 9			5 YR survival 80% * vs.* 77% SAVE *vs.* DOR Overall good results – SAVE better or equal to DOR Consider LVAD as ‘Bridge to restoration’ to remove urgency	Urgent cases
11	Suma	[34]	107	nonischemic dilated cardiomyopathy				7 *vs.* 61			OM fell from 42% to 15% after site selection	
12	Sartipy	[35]		10 yr DOR experience			8			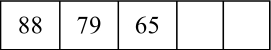	5 yr survival better in dyskinesia(80%) *vs.* akinesia (65%)	However DiDonato showed equivalent results in dyskinesia and akinesia
13	Williams	[36]		SVR in NYHA 4								
14	Menicanti	[7]			33 to 40		4.7				Survival 63% @ 10 yrs LVESV dec from 145 to 88. LVEDV dec from 211 to 142. NYHA dec from 2.7 to 1.6	MR + NYHA 2 + Diastolic dysfunction MVR/repair
15	Adams	[3]7		Impact of EF on SVR outcomes								EF<30% Mitral repair Age > 75 Advanced NYHA LVESVI > 80ml/m^2^
16	Patel	[38]		SVR in PHTN								
	SVR *vs.* VT											
17	Sartipy	[39]		SVR + endocardiectomy +/- cryoablation								
18	Dor	[4]	106	SVR + endocardiectomy +/- cryoablation							90% freedom from VT	
19	Mickleborough	[32]	108	SVR + endocardiectomy +/- cryoablation							freedom from VT, 99 & 94% @ 1 & 10 yrs	
20	Tulner	[40]		SVR + Mitral annuloplasty			12% @ 6 months					
21	Dor	[41]	835	SVR + Mitral annuloplasty			12%					
22	Di Donato	[42]		SVR + Mitral annuloplasty			19 *vs.* 26					
23	Qin	[43]	50	SVR + Mitral annuloplasty			5% late mortality					
24	Romano	[44]	>200	SVR + Mitral annuloplasty			5%					
25	Acker	[45]		SVR + Mitral annuloplasty			1.6%					
26	Hernandez	[46]	731	REAL WORLD APPLICATION			9.3% 8.9% @ 1 month			Reoperation 14%, stroke 3%, renal failure 8%, prolonged ventilation in 22%. Combined death or major complications - 34%.		Risk Factors for combined end point - age, female, creatinine ≥ 2 mg/dL, IDDM, recent MI, 3-vessel CAD, MR Emergency
27	Di Donato	[27]	74	Recent MI			5.4			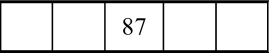		
28	Isomura	[47]										Type of procedure Emergency EDVI > 180 ml/m^2^

**Abbreviations:** No –number; Ref – reference; Pt no – patient number; EF - Pre/Post op – Ejection fraction preoperative and postoperative; LVESVI – left ventricular end systolic volume index; **LVEDVI** - left ventricular end diastolic volume index; OM – operative mortality’; CCF – congestive cardiac failure

## ABBREVIATIONS

AII: = Angiotensin II
BNP: = Brain natriuretic peptide
CABG: = Coronary artery bypass grafting
CAD: = Coronary artery disease
CHF: = Congestive heart failure
HF: = Heart failure
ICMPY: = Ischemic cardiomyopathy
IHD: = Ischemic heart disease
EF: = Ejection fraction
EVCPP: = Endoventricular circular patch plasty
DCM: = Dilated cardiomyopathy
GUSTO Trial: = Global Utilization of Streptokinase and Tissue Plasminogen Activator for Occluded Coronary Arteries
IABP: = Intra aortic balloon pump
ICD: = Implantable cardioverter defibrillator
LAD: = Left anterior descending artery
LCX: = Left circumflex artery
LV: = Left Ventricle
LMI: = Lateral wall myocardial infarction
LVA: = Left ventricular aneurysm
LVESVI: = Left ventricular end systolic volume index
LVESV: = Left ventricular end systolic volume
MI: = Myocardial infarction
MR: = Mitral regurgitation
MRI: = Magnetic resonance imaging
NE: = Norepinephrine
NT-pro-BNP: = N-terminal portion of proBNP
NYHA: = New york heart association
PHTN: = Pulmonary hypertension
PLV: = Partial left ventriculectomy
PRA: = Plasma renin assay
PTCA: = Percutaneous transluminal coronary angioplasty
REMATCH: = Randomized evaluation of mechanical assistance for the treatment of congestive heart failure
RESTORE Trial: = Reconstructive Endoventricular Surgery returning Torsion Original Radius Elliptical shape to the left ventricle
RCA: = Right coronary artery
RV: = Right Ventricle
SAVE procedure: = Septal Anterior Ventricular Exclusion
SAVE trial: = Survival and ventricular enlargement
STICH: = Surgical treatment for ischemic heart failure
SVR: = Surgical ventricular restoration
VT: = Ventricular tachycardia
TOAT: = Open artery trial
